# Association of the rs823144 variant of the RAB29 gene with the activity of lysosomal hydrolases in blood cells and risk of Parkinson’s disease

**DOI:** 10.18699/vjgb-25-89

**Published:** 2025-10

**Authors:** K.S. Basharova, A.I. Bezrukova, K.A. Senkevich, G.V. Baydakova, A.V. Rybakov, I.V. Miliukhina, A.A. Timofeeva, E.Yu. Zakharova, S.N. Pchelina, T.S. Usenko

**Affiliations:** Petersburg Nuclear Physics Institute named by B.P. Konstantinov of National Research Centre “Kurchatov Institute”, Gatchina, Russia Pavlov First Saint Petersburg State Medical University, St. Petersburg, Russia; Petersburg Nuclear Physics Institute named by B.P. Konstantinov of National Research Centre “Kurchatov Institute”, Gatchina, Russia Pavlov First Saint Petersburg State Medical University, St. Petersburg, Russia; Montreal Neurological Institute, McGill University, Montreal, Canada; Bochkov Research Centre for Medical Genetics, Moscow, Russia; N.P. Bechtereva Institute of the Human Brain of the Russian Academy of Sciences, St. Petersburg, Russia; Petersburg Nuclear Physics Institute named by B.P. Konstantinov of National Research Centre “Kurchatov Institute”, Gatchina, Russia N.P. Bechtereva Institute of the Human Brain of the Russian Academy of Sciences, St. Petersburg, Russia; Pavlov First Saint Petersburg State Medical University, St. Petersburg, Russia; Petersburg Nuclear Physics Institute named by B.P. Konstantinov of National Research Centre “Kurchatov Institute”, Gatchina, Russia Bochkov Research Centre for Medical Genetics, Moscow, Russia; Petersburg Nuclear Physics Institute named by B.P. Konstantinov of National Research Centre “Kurchatov Institute”, Gatchina, Russia Pavlov First Saint Petersburg State Medical University, St. Petersburg, Russia; Petersburg Nuclear Physics Institute named by B.P. Konstantinov of National Research Centre “Kurchatov Institute”, Gatchina, Russia Pavlov First Saint Petersburg State Medical University, St. Petersburg, Russia

**Keywords:** Parkinson’s disease, RAB29, lysosomal hydrolases, lysosphingolipids, LRRK2, болезнь Паркинсона, RAB29, лизосомные гидролазы, лизосфинголипиды, LRRK2

## Abstract

Recent genome-wide association studies have identified a link between the RAB29 gene and Parkinson’s disease (PD). The Rab29 protein encoded by RAB29 regulates leucine-rich repeat kinase 2 (LRRK2). Mutations in the LRRK2 gene increase its kinase activity and contribute to autosomal dominant forms of PD. Previous research has shown that altered LRRK2 kinase activity may correlate with the activity of lysosomal hydrolases and the concentration of sphingolipids. This study aimed to assess the association of the rs823144 variant in the promoter region of the RAB29 gene with PD risk, and to evaluate RAB29 expression, lysosomal hydrolase activity, and sphingolipid concentrations in the blood of PD patients. We screened the rs823144 variant of the RAB29 gene in a cohort of PD patients (N = 903) and controls (N = 618) using next-generation sequencing (NGS) and polymerase chain reaction (PCR) followed by restriction fragment length polymorphism analysis. The expression of the RAB29 gene was measured in peripheral blood mononuclear cells (PBMCs) using qPCR. We assessed the activities of lysosomal hydrolases (glucocerebrosidase (GCase), alpha-galactosidase (GLA), acid sphingomyelinase (ASMase), and galactosylcerebrosidase (GALC)) and the concentrations of sphingolipids (globotriaosylsphingosine (LysoGb3), sphingomyelin (LysoSM), and hexosylsphingosine (HexSph)) in blood using high-performance liquid chromatography with tandem mass spectrometry (HPLC-MS/MS). The RAB29 rs823144 C allele was associated with a reduced risk of PD in the Northwestern Russian population (OR = 0.7806, 95 % CI: 0.6578–0.9263, p = 0.0046), which is consistent with global data. However, no significant association was observed between the rs823144 C allele and RAB29 mRNA expression in PBMCs. Notably, the C allele was associated with increased GLA activity and decreased concentrations of LysoGb3 and LysoSM in the blood of PD patients. In conclusion, we demonstrate for the first time an association between the RAB29 rs823144 C allele and a reduced risk of PD in the Northwestern Russian population. Moreover, the RAB29 rs823144 C allele is associated with altered lysosomal enzyme activity and sphingolipid profiles, suggesting a potential role of RAB29 in sphingolipid metabolism relevant to PD pathogenesis.

## Abstract (ru)

Геномные исследования последних лет выявили ассоциацию гена RAB29 с болезнью Паркинсона (БП). Белок Rab29, кодируемый геном RAB29, – один из регуляторов богатой лейциновыми повторами киназы 2 (LRRK2). Мутации в гене LRRK2 ассоциированы с увеличением киназной активности LRRK2 и приводят к развитию аутосомно-доминантных форм БП. Недавно показано, что изменение киназной активности LRRK2 может быть связано с изменением активности лизосомных гидролаз и концентрации лизосфинголипидов. Цель данного исследования заключалась в оценке ассоциации rs823144 в промоторе гена RAB29 с БП с экспрессией гена RAB29, активностью лизосомных гидролаз и концентрацией лизосфинголипидов в крови при БП. В ходе исследования проведены скрининг варианта rs823144 гена RAB29 в группе пациентов с БП (N = 903) и в контроле (N = 618) с использованием методов массового параллельного секвенирования и полимеразная цепная реакция (ПЦР) с последующим рестрикционным анализом. Экспрессия гена RAB29 оценивалась в мононуклеарах периферической крови методом ПЦР в режиме реального времени. Активности лизосомных гидролаз (глюкоцереброзидаза (GCase), альфа-галактозидаза (GLA), кислая сфингомиелиназа (ASMase), галактозилцереброзидаза (GALC)) и концентрации лизосфинголипидов (глоботриаозилсфингозин (LysoGb3), сфингомиелин (LysoSM), гексозилсфингозин (HexSph)) оценивались в крови методом высокоэффективной жидкостной хроматографии с тандемной масс-спектрометрией (ВЭЖХ-МС/ МС). Аллель С rs823144 гена RAB29 ассоциирован с пониженным риском БП в северо-западной популяции Российской Федерации (ОШ: 0.7806, 95 % ДИ: 0.6578–0.9263, p = 0.0046), что соответствует мировым данным. Однако в ходе работы не выявлено ассоциации аллеля С rs823144 гена RAB29 с уровнем мРНК гена RAB29 в мононуклеарах периферической крови. В то же время носительство аллеля С rs823144 было ассоциировано с повышенной активностью GLA и сниженной концентрацией LysoGb3 в крови при БП. Таким образом, нами впервые показана ассоциация аллеля С rs823144 гена RAB29 с пониженным риском БП в северо-западной популяции Российской Федерации. Аллель С rs823144 ассоциирован с повышенной активностью GLA и сниженной концентрацией LysoGb3 в крови при БП. Полученные результаты позволяют предположить ассоциацию гена RAB29 с метаболизмом сфинголипидов.

## Introduction

Parkinson’s disease (PD) is a common, slowly progressive neurodegenerative disorder characterized by the loss of dopaminergic neurons in the substantia nigra (SN) of the brain (Lill, 2016). A central pathological mechanism in PD pathogenesis is the accumulation and aggregation of the alpha-synuclein protein in the SN. Although PD is primarily sporadic, approximately 15 % of patients report a positive family history. The molecular mechanisms underlying PD remain largely unclear; however, increasing evidence implicates lysosomal dysfunction as a key contributor to disease pathogenesis (Nechushtai et al., 2023). In particular, our group and others have demonstrated reduced lysosomal hydrolase activity and altered sphingolipid levels in the peripheral fluids of patients with idiopathic PD (Alcalay et al., 2015; Galper et al., 2022; Usenko et al., 2022). Additionally, changes in lysosomal enzyme activity and sphingolipid concentrations have been observed in PD cases associated with mutations in the LRRK2 gene, one of the most common monogenic forms of the disease (Alcalay et al., 2015; Usenko et al., 2023, 2024).

The LRRK2 gene encodes leucine-rich repeat kinase 2 (LRRK2), a multidomain protein implicated in Parkinson’s disease pathogenesis (Zimprich et al., 2004). A key group of LRRK2 substrates comprises small Rab GTPases, which are critical regulators of vesicular trafficking, particularly within the endolysosomal system (Steger et al., 2016; Wang et al., 2014). Dysregulated LRRK2 kinase activity disrupts the trafficking of lysosomal hydrolases to their proper destinations, thereby impairing lysosomal function (MacLeod et al., 2013; Ysselstein et al., 2019; Rivero-Ríos et al., 2020; Kedariti et al., 2022).

Among the LRRK2 substrates, Rab29 – encoded by the RAB29 gene – has attracted particular interest (Steger et al., 2016). Rab29 has been identified as a key upstream regulator of LRRK2, responsible for its activation (Liu et al., 2018; Madero-Pérez et al., 2018; Purlyte et al., 2018; Kuwahara Iwatsubo, 2020). Rab29 localizes to the membranes of lysosomes and the Golgi apparatus, where it recruits inactive cytoplasmic LRRK2 monomers and promotes their oligomerization into active dimers or tetramers (Purlyte et al., 2018; Zhu et al., 2023). The RAB29 gene is located within the PARK16 locus, which has previously been associated with reduced PD risk (Satake et al., 2009; Pihlstrøm et al., 2015; Nalls et al., 2019). A recent multi-trait analysis of genomewide association studies (MTAG) further confirmed the association between RAB29 and PD at both the transcriptomic and proteomic levels (Shi et al., 2024).

Several studies have identified variants in the promoter region of RAB29 that are associated with a reduced risk of Parkinson’s disease. These variants are thought to influence RAB29 gene expression levels (Gan-Or et al., 2012; Khaligh et al., 2017; Sun et al., 2021), potentially modulating the activation of LRRK2 and thereby affecting lysosomal hydrolase activity in PD

The aim of this study was to investigate the association of the rs823144 single nucleotide polymorphism (SNP), located in the promoter region of RAB29, with PD risk, RAB29 gene expression, lysosomal hydrolase activity – including glucocerebrosidase (GCase), α-galactosidase (GLA), galactocerebrosidase (GALC), and acid sphingomyelinase (ASMase) – and the concentrations of lysosphingolipids in the blood. The lysosphingolipids analyzed included hexosylsphingosine (HexSph), a mixture of glucosylsphingosine (GlcSph) and galactosylsphingosine (GalSph); lysosphingomyelin (LysoSM); and lysoglobotriaosylsphingosine (LysoGb3). These parameters were evaluated in both PD patients and healthy control subjects

## Materials and methods

Characteristics of the study groups. The study included 903 patients with sporadic PD and 618 control individuals matched for age and gender. All patients were recruited from the clinic of the N.P. Bechtereva Institute of the Human Brain of the Russian Academy of Sciences. The control group comprised individuals seen at the consultative and diagnostic center of the First St. Petersburg State Medical University named after academician I.P. Pavlov. To exclude PD and other neurodegenerative disorders, all control participants underwent neurological examination. Clinical and demographic characteristics of the study groups are presented in Table 1. No significant differences in age or gender distribution were observed between the groups (p > 0.05).

**Table 1.**
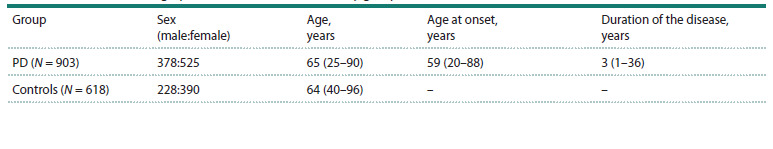
Clinical and demographic characteristics of the study groups Note. PD – Parkinson’s disease; data are presented as median (min–max).

All procedures involving human participants were conducted in accordance with the ethical standards of the National Research Ethics Committee and the 1964 Declaration of Helsinki and its later amendments or comparable ethical standards. Written informed consent was obtained from each participant prior to inclusion in the study. The study protocol was approved by the Ethics Committee of the First St. Petersburg State Medical University named after academician I.P. Pavlov (Protocol No. 275, dated September 04, 2023).

Genetic analysis. Two methods were used to genotype the rs823144 variant in the RAB29 gene: massively parallel sequencing (next-generation sequencing, NGS) and polymerase chain reaction (PCR) followed by restriction fragment length polymorphism (RFLP) analysis. Peripheral blood samples were collected from all study participants, and genomic DNA was extracted using phenol-chloroform extraction, as previously described (Maniatis et al., 1994).

NGS genotyping of the rs823144 variant in the RAB29 gene was performed in a subset of 521 PD patients and 420 control individuals using molecular inversion probes, as previously described (Rudakou et al., 2021). Sequencing was conducted on the Illumina NovaSeq 6000 SP PE100 platform. Sequence alignment was performed using the Burrows–Wheeler Aligner (BWA) with the hg19 human genome reference (Li, Durbin, 2009). Variant calling and post-alignment quality control were carried out using the Genome Analysis Toolkit (GATK, v3.8) (McKenna et al., 2010). Variants were filtered based on coverage depth and quality metrics; only those with a minimum read depth >30 and a quality score >20 were included in the analysis.

**Fig. 1.**
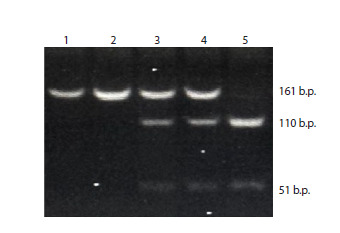
Electropherogram showing the results of genotyping of the rs823144 variant in the RAB29 gene 1, 2 – homozygote for the A allele (genotype AA), 3, 4 – heterozygote (genotype AC), 5 – homozygote for the C allele (genotype CC).

An additional 473 PD patients and 384 controls were screened for rs823144 using PCR followed by restriction analysis. Primer sequences were designed using Primer3 v. 0.4.0 (http://bioinfo.ut.ee/primer3-0.4.0/) (FOR: 5ʹ-CCCTGCA CGTGACGCTTG-3ʹ, REV: 5ʹ-GAATCCCAGTCAGCTC CTTACA-3ʹ). Restriction enzyme selection was performed using the NEBCutter tool (Vincze et al., 2003), and BstAC I was chosen for RFLP analysis (Fig. 1).

A total of 91 PD patients and 186 control individuals were genotyped for the rs823144 variant in the RAB29 gene using both PCR with restriction analysis and NGS. These overlaps were accounted for in subsequent statistical analyses

**Fig. 2.**
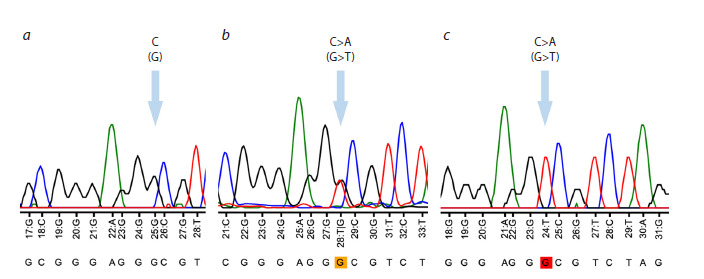
Graphical representation of Sanger sequencing results (electropherogram displaying the results of genotyping of the rs823144 variant of the RAB29 gene). a – homozygote for the C allele (genotype CC); b – heterozygote (genotype AC); c – homozygote for the A allele (genotype AA).

To validate the results obtained from both methods, a subset of samples was confirmed by Sanger sequencing using a Nanofor-05 genetic analyzer (Synthol, Russia). The Sanger sequencing data were visualized and analyzed using Tracy software (Rausch et al., 2020) (Fig. 2).

Evaluation of the relative expression level of the RAB29 gene in peripheral blood mononuclear cells of PD patients and controls. Peripheral venous blood samples were collected from PD patients (N = 30) and control individuals (N = 43). Peripheral blood mononuclear cells (PBMCs) were isolated by density gradient centrifugation using Ficoll-Paque PLUS (GE Healthcare) at 400g for 40 minutes, as described by Böyum (1968). The resulting mononuclear fraction was washed twice with phosphate-buffered saline (PBS; Biolot, St. Petersburg) and centrifuged at 3,000 rpm for 10 minutes. Total RNA was extracted from PBMCs using the RNeasy Mini Kit (Qiagen, 74104, USA). Complementary DNA (cDNA) was synthesized via reverse transcription using the RevertAid First Strand cDNA Synthesis Kit (K1622, Thermo Scientific, Lithuania).

The relative expression level of the RAB29 gene in PBMCs of PD patients (N = 30) and control individuals (N = 43) was quantified by real-time PCR using SYBR Green I as the intercalating dye. The housekeeping genes RPLP0 and GAPDH, which are constitutively expressed in PBMCs, were used as internal reference genes for normalization. Primer sequences were designed using the Primer3 v. 0.4.0 program (https://bioinfo.ut.ee/primer3-0.4.0) (FOR: 5ʹ-CGGTTTCACAGGTTGGACAG-3ʹ, REV: 5ʹ-CC CTTGGGTGGACAAAGACA-3ʹ). The relative mRNA level for each gene was calculated by comparing the threshold amplification levels ΔΔCt (Livak, Schmittgen, 2001).

Evaluation of lysosomal hydrolase activities and lysosphingolipid concentrations in peripheral blood of PD patients and controls. Peripheral venous blood samples were collected from PD patients and controls into EDTA tubes. To obtain dried blood spots, 40 μl of whole blood was applied to each spot on a filter paper test blank, after which the spots were allowed to air dry at room temperature for 2 hours and were then stored at +4 °C until extraction. Activities of four lysosomal hydrolases: glucocerebrosidase (GCase), α-galactosidase (GLA), galactocerebrosidase (GALC), and sphingomyelinase (ASMase), as well as the concentration of three lysosphingolipids: hexazyl sphingosine (HexSph) (a mixture of glycosyl sphingosine (GlcSph) and galactosyl sphingosine (GalSph)), lysosphingomyelin (LysoSM) and lysoglobotriaosyl sphingosine (LysoGb3) were estimated by high-performance liquid chromatography coupled with tandem mass spectrometry (HPLC-MS/MS) according to our previously published protocol (Pchelina et al., 2018).

Statistical data processing. Statistical analyses were performed using the R programming environment (version 4.0.5). Odds ratios (OR) with 95 % confidence intervals (CIs) were calculated using logistic regression models adjusted for age and gender. The nonparametric Mann–Whitney U-test was applied to compare the relative expression levels of the RAB29 gene, lysosomal hydrolase activities, and lysosphingolipid concentrations between study groups. To evaluate the association between the rs823144 variant of the RAB29 gene and lysosomal hydrolase activity, multiple linear regression analysis was conducted, adjusting for age, gender, and disease duration. Statistical significance was set at p < 0.05. Data are presented as median (min–max).

## Results

**Association between rs823144 of the RAB29 gene and PD risk**

Genotyping of the rs823144 variant in the RAB29 gene among PD patients and controls from the Northwestern Russian population revealed that the major allele is A. Hardy–Weinberg equilibrium (HWE) analysis confirmed that the genotype distributions in both groups were in equilibrium (p > 0.05). The genotype frequencies are summarized in Table 2. Logistic regression analysis demonstrated that the rs823144 variant is significantly associated with a reduced risk of Parkinson’s disease in this population (OR: 0.7806, 95 % CI: 0.6578–0.9263, p = 0.0046).

**Table 2.**
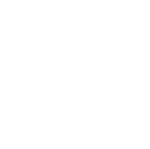
Frequencies of rs823144 genotypes and alleles in the study groups Note. PD – Parkinson’s disease; OR – odds ratio.

**Evaluation of the relative level of RAB29 gene mRNA in the mononuclear fraction of peripheral blood cells of PD patients and controls**

This study assessed for the first time the association between the rs823144 variant of the RAB29 gene and the relative expression level of RAB29 mRNA in peripheral blood mononuclear cells (PBMCs) from PD patients and controls. The median relative expression of RAB29 mRNA in PBMCs was 1.00 (0.22–1.75) in PD patients and 0.96 (0.13–1.79) in controls, with no statistically significant difference between groups ( p > 0.05). When stratified by rs823144 genotype, the relative RAB29 mRNA levels were as follows: in PD patients, AA genotype carriers had a median expression of 1.09 (0.–1.68), while carriers of AC and CC genotypes combined showed 1.13 (0.36–1.75); in controls, AA carriers had 1.05 (0.58–1.28) and AC + CC carriers had 0.97 (0.55–1.33). No significant association was found between the rs823144 genotype and RAB29 mRNA levels in either group ( p > 0.05).

**Association of the rs823144 variant of the RAB29 gene with the activity of lysosomal hydrolases and the concentration of lysosphingolipids in the peripheral blood of patients with PD and controls**

This study is the first to investigate the association of the rs823144 variant of the RAB29 gene with lysosomal hydrolase activities and lysosphingolipid concentrations in the blood of PD patients and controls. Patients with PD exhibited significantly increased GALC activity and decreased LysoSM concentrations compared to controls (p = 0.008 and p = 0.01, respectively) (Table 3). Stratification by rs823144 genotype revealed that carriage of the C allele of the rs823144 variant in the RAB29 gene was associated with increased GLA activity and decreased levels of LysoGb3 – a substrate of GLA – in PD patients (p = 0.038 and p = 0.022, respectively) (Table 3). These associations were confirmed by multiple linear regression adjusted for gender, age, and disease duration (GLA: β = 1.11, p = 0.024; LysoGb3: β = –0.23, p = 0.015) (Table 4). No significant differences were observed in GCase, GLA and ASMase activities, or HexSph and LysoGb3 concentrations when analyzing the combined groups of PD patients and controls, regardless of rs823144 genotype. Similarly, no genotype-dependent differences were detected in GCase, ASMase, GALC activities, or HexSph concentrations.

**Table 3.**
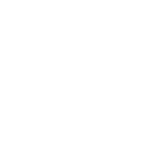
Lysosomal hydrolase activity and lysosphingolipid levels in the peripheral blood of PD patients and controls Note. PD – Parkinson’s disease; GCase – glucocerebrosidase; GLA – α-galactosidase; GALC – galactocerebrosidase; ASMase – sphingomyelinase; HexSph – hexasylsphingosine; LysoSM – lysosphingomyelin; LysoGb3 – lysoglobotriaosylsphingosine. * Compared with the combined control group; ** compared with PD patients with the AA genotype. Data are presented as median (min–max).

**Table 4.**
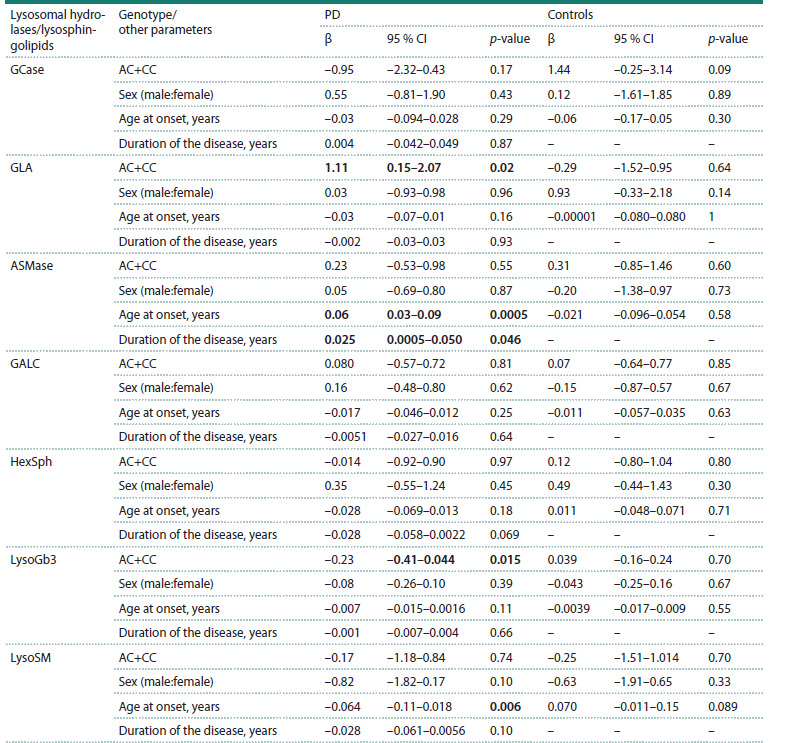
Lysosomal hydrolase activities and lysosphingolipid concentrations in the peripheral blood of patients with PD (regression analysis Note. PD – Parkinson’s disease; GCase – glucocerebrosidase; GLA – α-galactosidase; GALC – galactocerebrosidase; ASMase – sphingomyelinase; HexSph – hexasylsphingosine; LysoSM – lysosphingomyelin; LysoGb3 – lysoglobotriaosylsphingosine.

Notably, PD patients carrying the C allele exhibited decreased blood concentrations of LysoGb3 and LysoSM compared to the combined control group ( p = 0.045 and p = 0.015, respectively), whereas PD patients with the AA genotype showed increased LysoSM levels relative to controls ( p = 0.022). The increase in GLA activity and corresponding decrease in its substrate LysoGb3 were specific to carriers of C allele of the rs823144 variant in the RAB29 gene within the PD group and were not observed in controls

## Discussion

The RAB29 gene is one of five genes located within the PARK16 locus on chromosome 1q32, previously implicated in Parkinson’s disease risk (Simón-Sánchez et al., 2009; Tucci et al., 2010). Multiple association studies have identified the minor allele C of rs823144, positioned in the promoter region of RAB29, as protective against PD across various populations (Gan-Or et al., 2012; Xia et al., 2015; Khaligh et al., 2017; Sun et al., 2021). It has been hypothesized that this allele modulates transcription factor binding (Gan-Or et al., 2012; Khaligh et al., 2017).

In silico analyses suggest that the C allele of the rs823144 variant in the RAB29 gene abolishes the binding site for c- Ets-1 and introduces binding sites for p300, GATA-1, and Sp1, potentially enhancing RAB29 expression (Gan-Or et al., 2012). However, data from the GTEx database (https://www. gtexportal.org/) indicate that the CC genotype of rs823144 correlates with reduced RAB29 expression in whole blood and brain tissues ( p < 0.0001). In turn, increased expression of the RAB29 gene, encoding one of the main regulators of LRRK2 kinase, may potentially lead to impaired activation of this kinase. LRRK2, by phosphorylating Rab family proteins, regulates endolysosomal transport (Reczek et al., 2007; Wei et al., 2023). Dysregulation of LRRK2 activation can impair lysosomal hydrolase transport and function, contributing to PD pathogenesis

In our study of the Northwestern Russian population, we confirmed the association of the C allele of rs823144 with a reduced risk of PD, which is consistent with global findings (Gan-Or et al., 2012; Khaligh et al., 2017; Sun et al., 2021). Notably, we did not observe any association between the C allele of rs823144 genotype and RAB29 mRNA levels in peripheral blood mononuclear cells from either PD patients or controls.

Lysosomal dysfunction is widely recognized as a central mechanism in PD. Previous work by our group and others has demonstrated altered lysosomal hydrolase activity and sphingolipid metabolism in peripheral fluids and postmortem brain regions of PD patients (Alcalay et al., 2018; Nelson et al., 2018; Huebecker et al., 2019; Chang et al., 2022; Usenko et al., 2022, 2024). Specifically, we reported increased GALC activity and decreased LysoSM – a substrate of acid ASMase – concentration in PD peripheral blood (Usenko et al., 2022, 2024). In turn, disruption of lysosphingolipid metabolism, including through altered lysosomal hydrolase activity, may contribute to the aggregation of α-synuclein (Mazzulli et al., 2011; Marie et al., 2015).

Accumulating lysosphingolipids in neurons can stabilize neurotoxic alpha-synuclein oligomers (Battis et al., 2023). Prior brain autopsy studies revealed correlations between LysoGb3 isoform concentrations and pathological phosphorylated alpha-synuclein as well as an negative correlation of GLA activity with levels of α-synuclein phosphorylated at serine 129 – the pathological form of the protein that predominates in aggregates in PD (Nelson et al., 2018). Our novel findings linking the C allele of rs823144 to increased GLA activity and decreased LysoGb3 concentration, alongside elevated LysoSM levels in AA genotype carriers, underscore a potential role for RAB29 in sphingolipid metabolism in PD.

It should be noted that a marked reduction in GLA activity due to mutations in the GLA gene leads to a rare lysosomal storage disorder, Fabry disease. Notably, elevated LysoGb3 concentration is a risk factor for white matter lesions in Fabry disease. (Rombach et al., 2010). We have also observed LysoGb3 accumulation in neuronopathic mucopolysaccharidoses (Baydakova et al., 2020), suggesting that LysoGb3 elevation may not be exclusive to Fabry disease. Therefore, the association of the RAB29 rs823144 C allele with increased GLA activity and decreased LysoGb3 in PD patients may influence disease progression and clinical phenotype.

The present study has several limitations. The sample sizes of PD patients and the control group included in the experiment assessing the association of the RAB29 rs823144 variant with RAB29 mRNA levels were limited. The compared groups were population-wise heterogeneous. Furthermore, we did not directly assess the impact of rs823144 on LRRK2 kinase activity, which warrants investigation in future studies.

## Conclusion

This study demonstrates for the first time worldwide that the C allele of the rs823144 variant in the RAB29 gene, previously identified as protective against PD in other populations, is associated with a reduced risk of PD in the North-West region of Russia. Additionally, we report for the first time that carriage of the C allele correlates with increased GLA activity and decreased LysoGb3 concentration in the blood of PD patients.

These findings suggest a potential role for RAB29 in lysosphingolipid metabolism and imply that the rs823144 variant may influence the clinical course of PD. Further research is warranted to elucidate the relationship between the PARK16 locus, RAB29 gene, lysosphingolipid metabolism, and PD progression.

## Conflict of interest

The authors declare no conflict of interest.
